# Supplementary material for: The causal relationship between the human gut microbiota and pyogenic arthritis: a Mendelian randomization study
Source: Front Cell Infect Microbiol. 2024 Nov 26;14:1452480. doi: 10.3389/fcimb.2024.1452480 (PMC11629706; doi:10.3389/fcimb.2024.1452480)
Supplement: Supplementary file 1 [file DataSheet1.zip › mendelian test/ebi-a-GCST90027637.csv_leave_one_out.pdf]

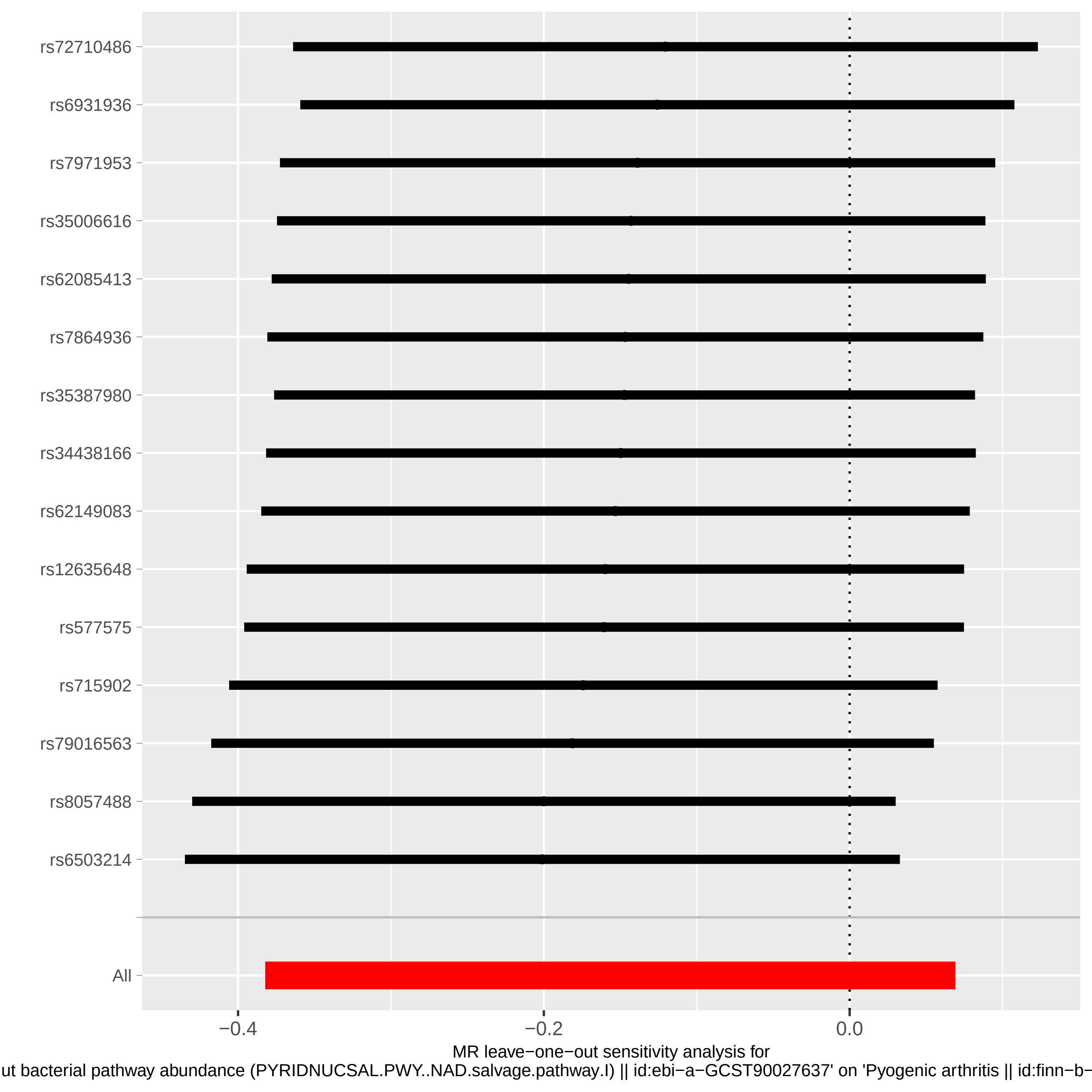
rs72710486
rs6931936
rs7971953
rs35006616
rs62085413
rs7864936
rs35387980
rs34438166
rs62149083
rs12635648
rs577575
rs715902
rs79016563
rs8057488
rs6503214
All
−0.4
−0.2
0.0
MR leave−one−out sensitivity analysis for
ut bacterial pathway abundance (PYRIDNUCSAL.PWY..NAD.salvage.pathway.I) || id:ebi−a−GCST90027637' on 'Pyogenic arthritis || id:finn−b−
